# Effect of Electrode Configuration on Nitric Oxide Gas Sensor Behavior

**DOI:** 10.3390/s150924573

**Published:** 2015-09-23

**Authors:** Ling Cui, Erica P. Murray

**Affiliations:** Institute for Micromanufacturing, Louisiana Tech University, Ruston, LA 71272, USA; E-Mail: lcu003@latech.edu

**Keywords:** NO_x_ sensors, diesel emissions, electrode configuration, porous YSZ electrolytes, impedance spectroscopy

## Abstract

The influence of electrode configuration on the impedancemetric response of nitric oxide (NO) gas sensors was investigated for solid electrochemical cells [Au/yttria-stabilized zirconia (YSZ)/Au)]. Fabrication of the sensors was carried out at 1050 °C in order to establish a porous YSZ electrolyte that enabled gas diffusion. Two electrode configurations were studied where Au wire electrodes were either embedded within or wrapped around the YSZ electrolyte. The electrical response of the sensors was collected via impedance spectroscopy under various operating conditions where gas concentrations ranged from 0 to 100 ppm NO and 1%–18% O_2_ at temperatures varying from 600 to 700 °C. Gas diffusion appeared to be a rate-limiting mechanism in sensors where the electrode configuration resulted in longer diffusion pathways. The temperature dependence of the NO sensors studied was independent of the electrode configuration. Analysis of the impedance data, along with equivalent circuit modeling indicated the electrode configuration of the sensor effected gas and ionic transport pathways, capacitance behavior, and NO sensitivity.

## 1. Introduction

Advancements in diesel fuel and engine technology continue to provide greater fuel efficiency and lower NO_x_ (*i.e.*, NO and NO_2_) emissions for diesel powered vehicles. NO_x_ emissions, which result from incomplete combustion of diesel fuel, are highly regulated by the US Environmental Protection Agency (EPA) as studies have shown that even concentrations as low as 20 ppm can be harmful to the environment and human health [1,2]. To address this issue, various diesel aftertreatment systems including, lean NO_x_ traps, selective catalytic reduction, fuel regeneration and diesel exhaust fluid (*i.e.*, urea), have been implemented to reduce NO_x_ to non-toxic gases [3,4]. NO_x_ sensors are crucial for verifying that such aftertreatment systems are working effectively. Current NO_x_ sensors are able to detect NO_x_ at concentrations down to approximately 10 ppm. However, it is expected that the lower emissions resulting from further development in diesel engine technology, as well as increasingly stringent EPA standards, will require sensors that are capable of monitoring NO_x_ concentrations as low as 1 ppm.

Yttria-stablized ziriconia (YSZ) has been widely used in NO_x_ sensors as the solid-state electrolyte has a high tolerance for the harsh exhaust gas environment [5]. The electrolyte is typically accompanied with noble metal (or metal oxide) electrodes; and, NO_x_ sensing is understood to take place at the electrolyte/electrode interface. Porous YSZ electrolytes have recently be shown to promote greater NO_x_ sensitivity [6]. Studies have shown that the NO_x_ sensor response is also dependent upon various properties related to the sensing electrode. For example, the material composition, microstructure, surface area, and geometry of the sensing electrode can affect the sensitivity, selectivity and response time of the NO_x_ sensor [7,8,9,10,11,12]. Fewer studies have considered the role of electrode configuration on NO_x_ sensing behavior. Interestingly, this aspect of the sensing electrode design directly impacts the electrochemical response and reliability of the NO_x_ sensor [13]. Further knowledge of the role of the sensing electrode configuration can contribute to a more complete understanding of the electrode properties on gas sensing.

Although NO and NO_2_ are present in the diesel exhaust stream, NO tends to be more stable at elevated temperatures and is significantly more abundant (e.g., over 90% at 600 °C) due to thermodynamic conversion of NO_2_. Hence, this study concentrates on the impedancemetric response to NO of porous YSZ electrolyte sensors with different electrode configurations. The influence of electrode configuration on gas and ionic transport pathways, reactions at the electrode/electrolyte interface, and rate-limiting mechanisms is discussed with respect to the sensor response to NO.

## 2. Experimental Section

**Figure 1:**
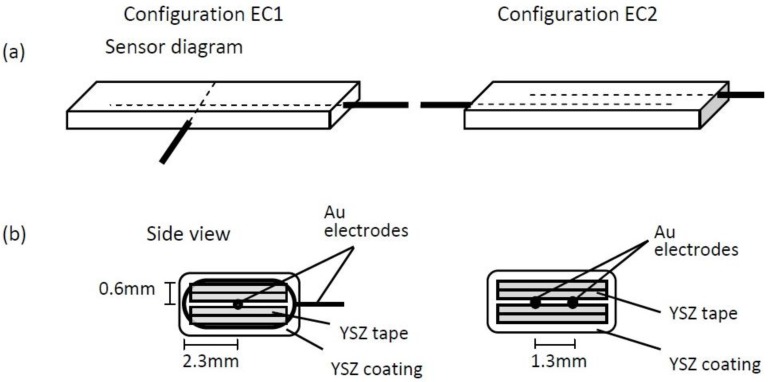
Diagrams of the NO sensors with electrode configurations (**a**) EC1 and EC2; (**b**) Cross sectional view illustrating the placement of the Au electrodes within the YSZ tape layers composing the sensor.

The NO sensors were composed of tape cast YSZ for the electrolyte and dense Au wire (0.25 mm diameter, Alfa Aesar) electrodes. The YSZ tape was prepared from YSZ powder (8 mol% yttria-stabilized zirconia, Tosoh Corp.) that was ball milled with B-98 Butvar binder, phosphate ester for the dispersant, dipropylene glycol benzoate as the plasticizer, along with the solvents ethanol and xylene. The doctor blade method was used to cast this mixture into thin sheets, referred to as tapes. The YSZ tapes were used to construct two types of sensors with different electrode configurations. Sensors with electrode configuration 1 (EC1) had a Au wire electrode embedded between four layers of YSZ tape, and a second Au wire electrode that was wrapped around this arrangement. A slurry made from dissolving YSZ tape into ethanol was applied as a coating over the entire sensor assembly (see Figure 1). Sensors with electrode configuration 2 (EC2) were made by embedding two parallel Au wire electrodes between four layers of YSZ tape. Similarly, YSZ slurry was applied over the outer surfaces of the tapes to complete the sensors. Both types of sensors were fired at 1050 °C for 1 h. Prior studies by the authors have found that this firing condition produces an electrolyte microstructure with a porosity of about 46% that promotes gas diffusion and electrochemical NO_x_ sensing reactions [9]. After firing, the rectangular shaped NO_x_ sensors were approximately 10 mm × 5 mm with a thickness of about 1.5 mm.

Impedance spectroscopy experiments were carried out using a Gamry Reference 600 in order to characterize the electrical response to NO for EC1 and EC2 sensor. The experiments were performed within a quartz tube that was placed in a furnace such that data could be collected for operating temperatures ranging from 600 to 700 °C. The sensors were exposed to gas environments containing 1%–18% O_2_ with 0–100 ppm NO where the total flow rate was 100 sccm. The gas composition was managed using thermo mass flow controllers that were connected to four certified gas cylinders (NO, NO_2_, N_2_, and air) with N_2_ as the balance gas. Data were collected over a frequency range of 1 Hz to 1 MHz with an excitation voltage of 50 mV. As this excitation voltage was within the linear voltage regime of the sensor equivalent circuit modeling could be performed with high accuracy to analyze the impedance results.

## 3. Results and Discussion

### 3.1. Impedance Response

**Figure 2:**
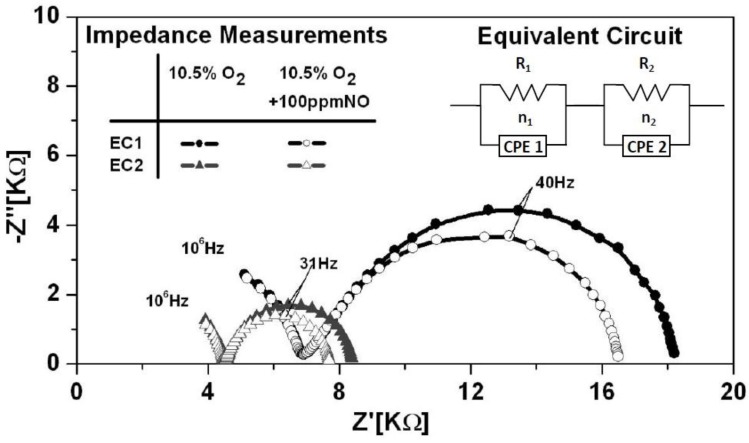
Typical Nyquist plot showing the impedance response of EC1 and EC2 sensors at an operating temperature of 650 °C, in 10.5% O_2_ with and without NO present. The equivalent circuit used to model the data is shown in the upper right corner of the plot. The model that fit the data is described by the solid lines.

Impedance measurements were carried out in order to determine the effect of electrode configuration on the electrical response of the NO_x_ sensors. Figure 2 shows typical impedance data collected for EC1 and EC2 sensors at an operating temperature of 650 °C in the presence of 10.5% O_2_ with and without 100 ppm NO. The impedance response for 100 ppm NO_2_ was very similar to the response measured for 100 ppm NO. Similar results were observed in previous studies [9]. Thermodynamic conversion of NO_2_ to NO causes the vast majority (over 90%) of NO_2_ to convert to NO at temperatures over 600 °C. Thus, data presented here concentrates on the NO response. The data was measured in triplicate for each testing condition to insure that stable, reproducible data was collected. The impedance data indicated two distinct arcs for both the EC1 and EC2 sensors. The incomplete high frequency arc (HFA) resulted from the frequency limit of the Gamry Reference 600. Bulk electrolyte properties associated with the porous YSZ microstructure, such as oxygen ion conductivity are described by the high frequency response. More importantly, the impedancemetric NO_x_ sensor response is primarily described by electrode reactions, which are presented in the low frequency regime. The impedance response for EC1 sensors with the Au wire loop and embedded electrodes was substantially larger than the response of the EC2 sensors with parallel Au wire electrodes. In general, the impedance response of NO_x_ sensors depends upon the material properties, molecular and electronic transport, as well as the following electrochemical reduction/oxidation reactions:

(1) $$\frac{1}{2}O_{2}+2e^{-}↔O^{-2}$$

(2) $$NO_{2}+2e^{-}↔NO+O^{-2}$$

These reactions occur along the triple phase boundary (TPB) where the electrode, electrolyte and gas species come into contact with each another. The low frequency impedance response is influenced by TPB Reactions (1) and (2) [6,7]. Since the sensors are composed of the same materials, it is likely that similar reactions occurred at each sensor. However, the pathways associated with those reactions may have differed. The tortuous microstructure of the porous electrolyte and, in particular, the distance between the electrodes affects the molecular and ionic transport within the sensors. Figure 1 shows the distance between the wire loop and embedded electrodes in the EC1 sensors varied from approximately 0.6–2.3 mm; whereas, the parallel electrodes for EC2 sensors were about 1.3 mm apart. The variation in distance between the electrodes within EC1 sensors caused NO_x_ and O_2_ gas species, as well as oxygen ions to travel longer distances through the tortuous pathways of the porous electrolyte in order to participate in TPB reactions. Similar observations have been made in solid oxide fuel cell (SOFC) studies where larger impedance measurements are reported for SOFCs composed of a thick film *versus* thin film electrolyte. [14,15] The thick film electrolyte results in a greater distance between the electrodes, thereby, creating a longer pathway for oxygen ion transport through the electrolyte. As a result greater ohmic losses occur, which contribute to higher impedance values. Thus, the longer pathways for gas and oxygen ion transport within the EC1 sensors most likely contributed to the larger impedance.

Equivalent circuit modeling with Gamry EIS300 software was used to simulate the impedance measurements and further analyze the electrical response of the sensors under various operating conditions. The equivalent circuit model that best fit the sensor impedance results was, (R_1_CPE_1_) (R_2_CPE_2_), as shown in Figure 2 for both sensor configurations. The resistor, R_1_, was associated with the HFA and interpreted as the ionic transport resistance of the porous YSZ electrolyte. Other studies have shown that the HFA dependents upon the porosity of the electrolyte as the tortuous pathways within the porous microstructure impede the flow of oxygen ions [9,16]. The low frequency arc (LFA) resistance, R_2_, described the interfacial resistance associated with TPB reactions. Such reactions include adsorption, dissociation, and diffusion of gases, as well as charge transfer, which can contribute to the electrode resistance. Further details on electrode reactions are discussed in follow sections. The equivalent circuit model accounted for the slight suppression of the arcs with constant phase elements, CPE_1_ and CPE_2_, which described the non-ideal capacitance behavior of the electrolyte and electrodes, respectively. Surface defects, interfacial reactions, as well as the morphology of the sensor components, can cause the time constant for various reactions to differ resulting in non-ideal capacitance behavior [6]. Table 1 shows the fitted values associated with the equivalent circuit model shown in Figure 2. The goodness of fit (*i.e.*, Chi squared) describes the quality of the fit. The errors associated with the fitting parameters were 1%–4% and 5%–6% where the goodness of fit was on the order of 10^−6^ and 10^−4^, respectively. The impedance data collected under the various operating conditions were fit using the same model and had goodness of fit values within this range.

**Table 1:** Equivalent circuit fitting parameters are shown for sensor impedance data collected at 650 °C.

Sensor	High Frequency Arc			Low Frequency Arc						Goodness of Fit
	10.5% O_2_			10.5% O2			10.5% O_2_ + 100 ppm NO			
	CPE_1_ (F)	n_1_	R_1_ (kΩ)	CPE_2_ (F)	n_2_	R_2_ (kΩ)	CPE_2_ (F)	n_2_	R_2_ (kΩ)	
EC1	1.11 × 10^−10^	0.9	7.01	0.92 × 10^−6^	0.8	11.65	0.89 × 10^−6^	0.9	9.87	2.10 × 10^−4^
EC2	0.75 × 10^−10^	0.9	4.54	2.22 × 10^−6^	0.9	3.90	2.24 × 10^−6^	0.9	3.23	2.90 × 10^−6^

**Figure 3:**
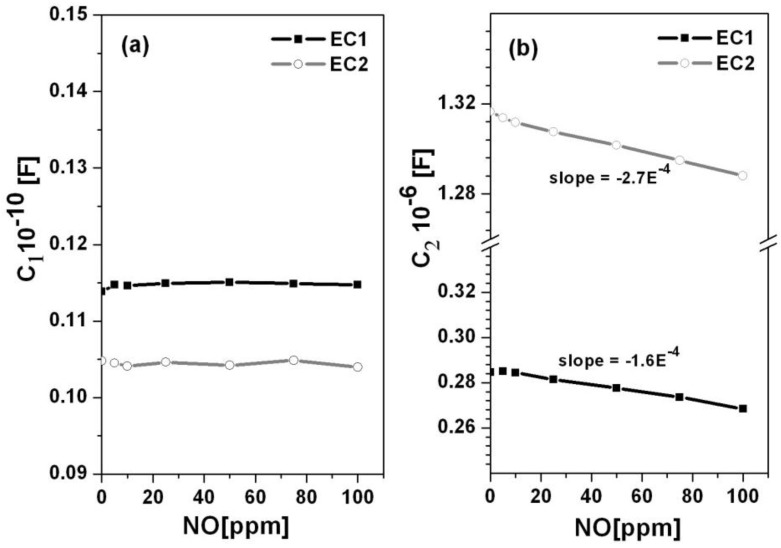
The capacitance *versus* NO concentration is shown for the (**a**) high and (**b**) low frequency arcs associated with the EC1 and EC2 sensors.

The fitted values for the CPE data generated for EC1 and EC2 sensors were used to calculate the capacitance, C, associated with the high and low frequency sensor response according to the following equation:

(3) $$C=\;\frac{{\left[(R)(CPE)\right]}^{\frac{1}{n}}}{R}$$

where R was the resistance associated with the low frequency impedance arc, and *n* describes the deviation from pure capacitance behavior. In the case where *n* = 1, the constant phase element represents a pure capacitor. Figure 3 shows the capacitances, C1 and C2, with respect to NO concentration for the EC1 and EC2 sensors, respectively. The EC2 sensors had higher capacitance values in comparison to the EC1 sensors. Higher capacitance has been associated with increased oxygen coverage at the Au/YSZ interface [17]. If oxygen arrived at this interface faster than it could participate in TPB reactions, then the shorter path length between the embedded parallel electrodes in the EC2 sensors may have enabled oxygen accumulation at the Au/YSZ interface. As for the EC1 sensors, the greater distance between the wire loop and embedded electrodes may have caused oxygen transport to occur over a longer time period such that the rate of oxygen arriving at the Au/YSZ interface coincided more closely with TPB reaction rates. In such a case, oxygen arriving at the Au/YSZ interface would more immediately participated in interfacial reactions, such that limited accumulation occurred. This suggests that the rate of oxygen transport through the porous YSZ electrolyte in EC1 sensors agreed more closely with the rate of interfacial reactions. The data in Figure 3 also indicates that as the concentration of NO increased, the capacitance decreased, which suggests that oxygen coverage diminished as NO occupied more sites along the TPB.

### 3.2. NO Sensitivity

**Figure 4:**
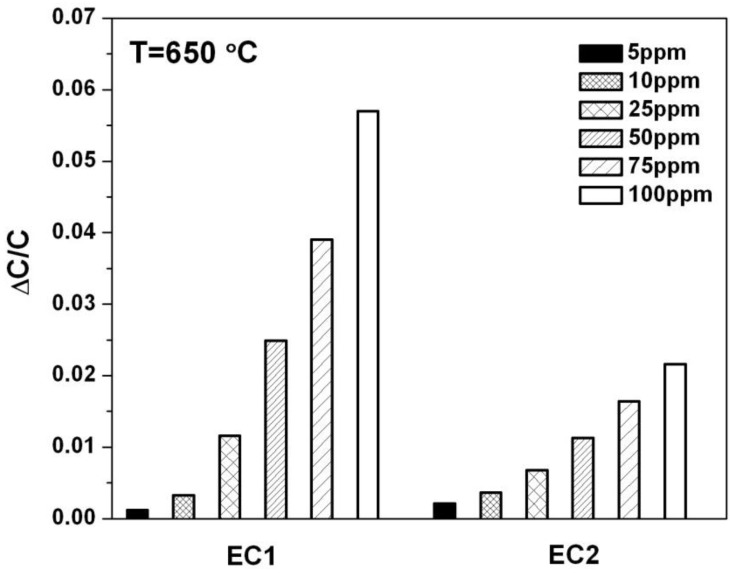
The change in capacitance is plotted for EC1 and EC2 sensors for NO concentrations ranging from 5 to 100 ppm.

Various methods have been used to assess NO sensor sensitivity. The operating conditions as well as material properties of the sensor components can significantly influence the degree of sensitivity observed by a given method. [18] Figure 4 shows NO sensitivity for EC1 and EC2 sensors in terms of the change in capacitance, ΔC/C, where $\text{Δ}C/C=[(C_{O2}-C_{NO})/C_{O2}]$ [19]. The terms C_O2_ and C_NO_ were the capacitance values calculated from fitted data for the LFAs according to Equation (3). C*_O2_* was the baseline capacitance that was determined when only 10.5% O_2_ was exposed to the sensor. The capacitance, C*_NO_*, was calculated based on the presence of a specific concentration of NO in the gas surrounding the sensor. The data in Figure 4, indicates the sensitivity to NO was significantly greater for EC1 *versus* EC2 sensors. These findings along with the capacitance data in Figure 3 suggest that lower oxygen coverage at the electrode/electrolyte interface in EC1 sensors may have allowed more NO reactions to take place at the TPB, which contributed to greater NO sensitivity.

**Figure 5:**
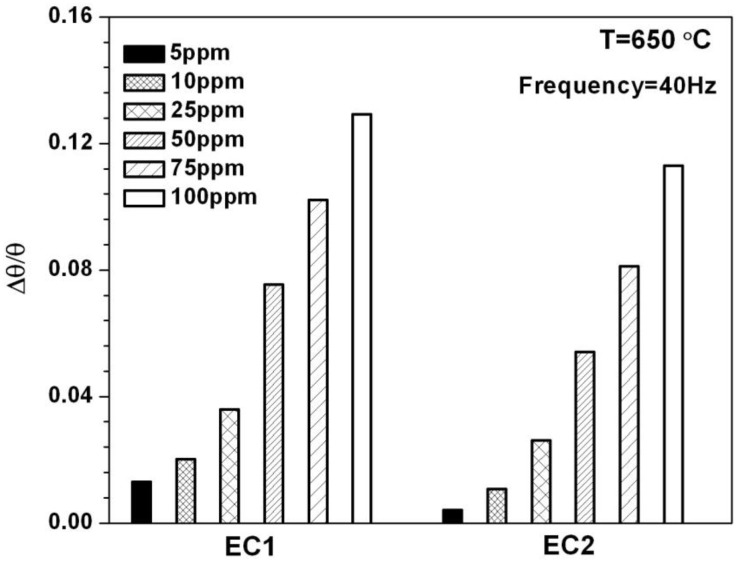
The change in the angular phase angle is plotted for EC1 and EC2 sensors for NO concentrations ranging from 5 to 100 ppm.

The phase angle *θ*, is a frequency dependent parameter that also can be used to evaluate the impedancemetric NOx sensor response. The relationship between the phase angle and the impedance is given by the equation $\theta =tan^{-1}(Z^{\prime \prime }/Z^{\prime })$, where $Z^{\prime \prime }$ and $Z^{\prime }$ are the imaginary and real components of the impedance, respectively. NO sensitivity can be determined by calculating the percent change in the phase angle response, Δθ/θ, for a specific operating frequency when the sensor is exposed to NO. The percent change in phase is defined as $\text{Δ}\theta /\theta =[(\theta_{O2}-\theta_{NO})/\theta_{O2}]$, where *θ_O2_* represented the baseline phase when only 10.5% O_2_ was present and *θ_NO_* described the phase response for a specific concentration of NO. Figure 5 shows the NO sensitivity for EC1 and EC2 sensors based on the phase response at 40 Hz. This data agrees with the sensitivity data presented in Figure 4, as EC1 sensors demonstrated greater NO sensitivity in comparison to EC2 sensors. Further comparison between Figure 4 and Figure 5 indicates NO sensitivity based on Δθ/θ is greater than the sensitivity associated with ΔC/C, particularly for the EC2 sensors. The phase response data also shows a higher sensitivity at lower NO concentrations, specifically 5 and 10 ppm. It is important to note that NO sensitivity based on Δθ/θ is dependent upon the sensor operating frequency. In general, the higher the operating frequency the more rapid the sensor response becomes to changes in NO concentration; however, NO sensitivity generally decreasing as the operating frequency increases. In this study, the Δθ/θ data collected at 40 Hz was determined to be the most suitable operating frequency with respect to such tradeoffs.

### 3.3. Oxygen and Temperature Dependence

**Figure 6:**
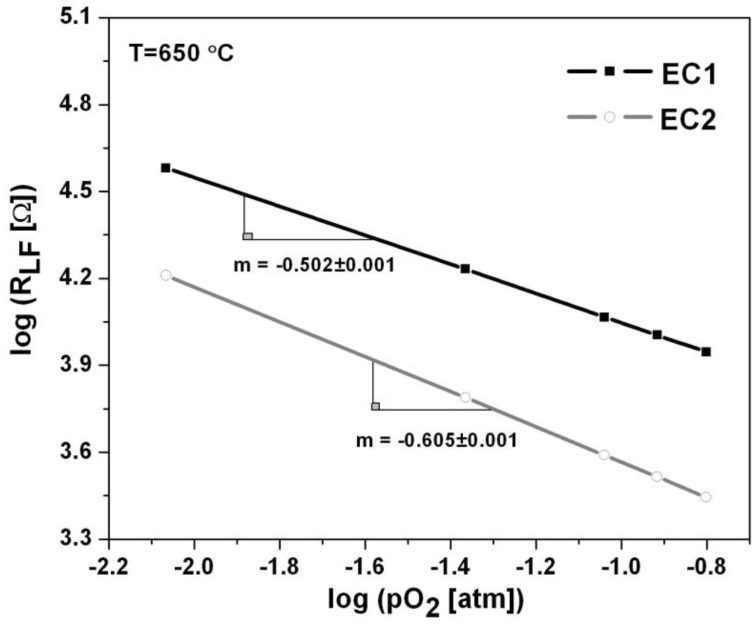
The oxygen partial pressure dependence of the EC1 and EC2 sensors is shown for data collected at 650 °C for oxygen concentrations ranging from 1% to 18%.

The oxygen partial pressure and temperature dependence of the sensors can further understanding of potential rate limiting mechanisms and kinetic reactions that influence sensor behavior. The oxygen partial pressure dependence was determined for the low frequency arcs associated with the EC1 and EC2 sensors according to the power law relationship, $R\propto {\left(pO_{2}\right)}^{m}$, where the slope, m, describes the rate limiting step. As shown in Figure 6, a dependence of $R_{1}\propto {\left(pO_{2}\right)}^{-0.50}$ and $R_{2}\propto {\left(pO_{2}\right)}^{-0.62}$ was observed for EC1 and EC2 sensors, respectively. Studies concerning oxygen partial pressure dependence have reported that slope values corresponding to −0.5 indicate electrode surface reactions involving dissociative adsorption or atomic diffusion of oxygen as the rate limiting mechanism [20]. As the slope becomes closer to −1 gas diffusion has been considered as rate limiting [20]. The more negative slope dependence, m = −0.62, for the EC2 sensors seems to suggest gas diffusion contributed to the rate limiting steps. It is possible that the diffusion depth to the electrode/electrolyte interface within the EC2 sensors limited gas diffusion. The EC2 sensors were composed of parallel electrodes that were embedded approximately 0.75 mm within in the electrolyte; whereas, the wire loop electrode around the EC1 sensors had a thin electrolyte coating of about 0.15 mm in thickness. Thus, O_2_ and NO gases traveled a shorter path to a TPB within the EC1 sensors, in comparison to EC2 sensors where the diffusion depth was greater.

**Figure 7:**
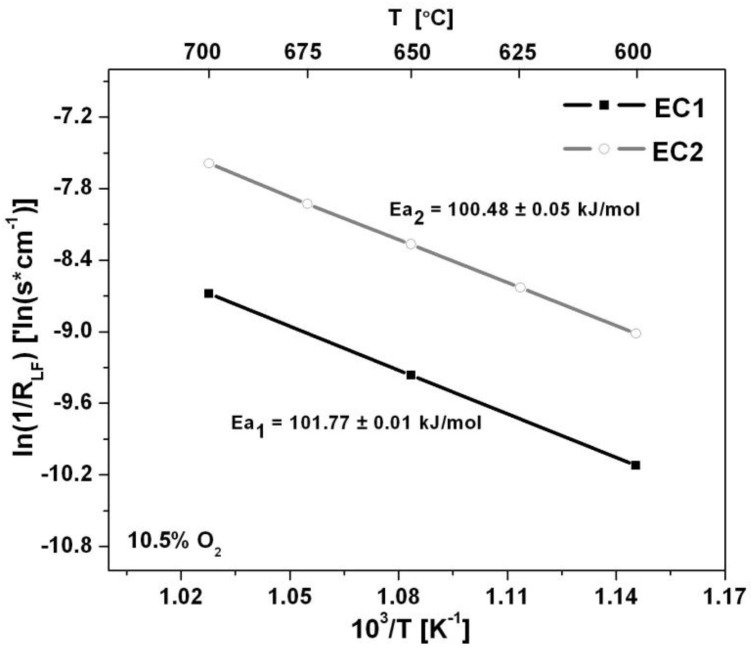
The temperature dependence is described by the Arrhenius plot for the EC1 and EC2 sensors along with corresponding activation energies.

Arrhenius plots shown in Figure 7 describe the temperature dependence of the sensors. The activation energies calculated from the slope of the Arrhenius plots were very similar for each electrode configuration as Ea_1_ = 101.77 ± 0.01 kJ/mol and Ea_2_ = 100.48 ± 0.05 kJ/mol for EC1 and EC2 sensors, respectively. The activation energy describes the temperature dependence of the electrode/electrolyte system. As the operating temperature increased the activation energy decreased suggesting that interfacial reactions were able to proceed more readily. The similar behavior described by the activation energy for the EC1 and EC2 sensors indicates a negligible relationship between the electrode configuration and temperature dependence of the sensors.

## 4. Conclusions

The effect of electrode configuration on NO sensitivity was investigated using Au/YSZ/Au sensors over an operating temperature range of 600–700 °C. The electrode configuration seemed to primarily effect the transport distances for NO and O_2_ gas species and oxygen ions. The distance between the Au wire loop and embedded electrodes in the EC1 sensors resulted in longer pathways for gas and ionic transport between the electrodes, in comparison to the parallel electrodes within EC2 sensors. As a result the total impedance was larger for EC1 in comparison to EC2 sensors. The equivalent circuit model was the same for both sensor configurations; however, the fitted data results differed. In particular, the calculated capacitance values determined from the fitted data suggested that the oxygen coverage at the electrode/electrolyte interface was significantly lower for the wire loop electrode configuration of EC1 sensors. It is possible that the lower oxygen coverage allowed for higher NO coverage at the electrode/electrolyte interface, thereby, contributing to greater NO sensitivity for the EC1 sensors. Analysis of the oxygen partial pressure dependence suggested that dissociative adsorption or atomic diffusion were likely rate-limiting mechanisms for each sensor type. Although the electrolyte porosity was the same for both types of sensors, gas diffusion appeared to be an additional rate-limiting step for EC2 sensors as the gas diffusion path to TPBs was greater. The electrode configuration did not have a significant effect on temperature dependence of the sensors as the activation energies for the EC1 and EC2 sensors were similar.
